# Gender differences in the impact of sleep duration on common mental disorders in school students

**DOI:** 10.1186/s12889-020-8260-5

**Published:** 2020-01-31

**Authors:** Beatriz Tosé Agathão, Claudia Souza Lopes, Diana Barbosa Cunha, Rosely Sichieri

**Affiliations:** Department of Epidemiology, Institute of Social Medicine, State University of Rio de Janeiro (UERJ), 524 São Francisco Xavier St., Maracanã, Rio de Janeiro, 20550-900 Brazil

**Keywords:** Mental health, Mental disorders, Children, Adolescents, Sleep duration, Sleep hours, Insufficient sleep

## Abstract

**Background:**

Common mental disorders (CMD) in children and adolescents can be initial and non-specific manifestations of more serious mental diseases and often persist into adulthood. Therefore, early detection is important, as is the identification of the factors that impact development. Insufficient sleep represents one of the most common and potentially remediable risks to mental health in children and adolescents for whom chronic sleep loss has become normal. This study aims to investigate the influence of sleep duration on CMD in schoolchildren and adolescents.

**Methods:**

The data for this longitudinal study were derived from the community trial Parents, Students, Community Health Agents and Teachers for Healthy Eating (PAAPAS) in 2016 (*N* = 2743), with fifth and sixth graders from 18 public schools of Duque de Caxias, Rio de Janeiro, Brazil. CMD were assessed by the General Health Questionnaire (GHQ-12) at baseline and at a 9-month follow-up. Sleep duration was evaluated at baseline and was categorized as short, adequate or long according to age group. The effect of sleep duration on common mental disorders was analysed by sex using linear mixed-effects models.

**Results:**

At baseline, the frequency of CMD was 33.2% and was higher in boys than in girls and higher among students with short sleep duration. There was an increase in the CMD score over time among girls with short sleep duration (*p < 0.01*). Among boys, a similar trajectory of the CMD score was observed in the subgroups with short and long sleep duration, but there was a significant reduction in the subgroup with long sleep duration (*p = 0.05*).

**Conclusion:**

Changes in common mental disorder patterns differ according to sex, and short sleep duration seems to be problematic for students’ mental health. The promotion of health strategies that involve the family and school environment, such as later school hours, could help change this scenario.

## Background

Problems related to mental health in childhood and adolescence constitute an important part of the global burden of diseases (10–20%) and are relevant because they represent one of the main causes of health and developmental losses in this age group. Additionally, they are long-lasting and often persist into adulthood [1]. Non-psychotic mental disorders affect approximately 20–30% of the general population and are known as common mental disorders (CMD) [2, 3]. Marked mainly by depressive symptoms, anxiety and various somatic complaints, they are associated with sociodemographic and lifestyle factors such as economic status, alcohol and drug consumption, sedentary behaviour, physical inactivity and, more recently, sleep disorders [4–8].

Sleep problems and sleep-related complaints are frequent among individuals of different age groups [9, 10]. Studies show a tendency of adolescents to present irregular periods of sleep, reducing the amount and quality of sleep over time due to the biological, social and academic changes typical of this phase. The definition of what is insufficient or excessive sleep is still controversial, and there are no definitive conclusions about what is healthy sleep since there are individual aspects such as genetic characteristics that determine this need [11, 12]. However, some recommendations on sleep duration have been proposed; for example, the American Academy of Sleep Medicine (AASM), which focuses on childhood and adolescence, suggests that children between 6 and 12 years old should sleep from 9 to 12 h, and adolescents between 13 and 18 years old should sleep from 8 to 10 hours [13, 14]. Although most children and adolescents require approximately 9 h of sleep per night, fewer than 8% of high school students achieve this amount, and this proportion decreases as school grade level increases [15].

Evidence suggests that sleep duration plays an important role in health outcomes. In a recent publication, Czeisler [16] related the main findings of several studies on the health consequences of changes in sleep quality, duration and time. The author points out that sleep deficiency critically affects physical and mental health, performance and safety, and a period of only one to two weeks of reduced hours of sleep leads to increased attention deficit and mood disorders. Poor sleep may lead to excessive sleepiness during the day, which is one of the main consequences of sleep disturbances and has a close relation with declining school performance and with a negative perception of the quality of life among adolescents [17]. It has also been observed that girls are more vulnerable to insufficient sleep than boys, exhibiting more anxious and depressive symptoms and mood deficits [18–20].

In some studies [21–24], insufficient sleep in young people is associated with a wide variety of adverse outcomes, from poor mental health to behavioural problems and lower academic performance, but much of this evidence is made up of cross-sectional studies, and few studies have investigated the direction of this relationship. In children and adolescents, CMD can be initial and non-specific manifestations of more serious mental disorders; therefore, early detection and the identification of factors that impact development are important [8]. Additionally, insufficient sleep represents one of the most common, important and potentially remediable risks to mental health in children and adolescents for whom chronic sleep loss has become normal [25, 26]. The purpose of this study is to evaluate the influence of sleep duration on CMD in schoolchildren and adolescents.

## Methods

### Study design and participants

Data for this prospective study were derived from the trial labelled PAAPAS, which stands for “Parents, Students, Community Health Agents and Teachers for Healthy Eating”, a randomized community-controlled trial conducted in 2016 in a sample of fifth and sixth graders from 18 public schools in the municipality of Duque de Caxias, Rio de Janeiro, Brazil.

PAAPAS’s purpose was to reduce excessive weight gain among adolescents by combining primary intervention at schools with secondary intervention through trained community health agents (CHAs). The primary intervention in the school, conducted by trained teachers, provided the overall basis for a healthy lifestyle, including food intake, physical activity, and non-sedentary habits. For adolescents diagnosed with excessive weight, household activities were provided as additional motivation to change these behaviours. A complete description of each intervention and details on the sample size and randomized procedures are available in the PAAPAS protocol [27].

The municipality of Duque de Caxias [28] (population, 842,686) is located in the metropolitan area of Rio de Janeiro, 27 km from the state capital. It is one of the poorest areas in Brazil. For the PAAPAS study, two of four districts in Duque de Caxias were included, and from 45 municipal schools, 18 schools (9 interventions and 9 controls) with fifth and sixth grade classes were selected. For this study, data from schoolchildren from all the selected schools were used, regardless of the group to which they belonged.

The Ethics Committee of the Institute of Social Medicine (State University of Rio de Janeiro, Brazil) approved the protocol. Written informed consent was obtained from all the participants’ parents. Students with physical or mental disabilities and girls who were pregnant or lactating were considered ineligible and were not included in the study.

### Measurements

All anthropometric measures were collected, and the questionnaire was administered at the beginning of the school year and at the end of the school year, with 9 months between the collection dates. Students completed a structured questionnaire using personal digital assistants (PDAs) under the supervision of field researchers.

Common mental disorders (the outcome measure) were evaluated using the Brazilian version of the General Health Questionnaire – 12 items (GHQ12) [29] at both baseline and follow-up. The GHQ12 is a self-reported instrument to track non-psychotic mental disorders. It was created to identify symptoms of depression and anxiety, inability to deal with habitual situations and lack of self-confidence, feelings commonly found in the general population. Each GHQ item has four response options and a reference period of two weeks before completing the questionnaire. The questionnaire had previously been validated for the Brazilian population and has a structured psychiatric interview as a gold standard, with good psychometric properties [30]. The Brazilian version of the GHQ has shown a sensitivity of 76% and specificity of 82% at the cut-off point of 4/5 [31]. To observe the individual CMD frequency scores, items were recoded as “absent” or “present” (0 or 1, respectively) and then summed. The cut-off point of 4/5, used by Fortes [32] for the detection of severe-intensity mental disorders, was applied. To observe a severity spectrum, a continuous variable, ranging from 0 to 36 potential points, was composed after recoding each item on a scale of 0 to 3 points.

Sleep duration was assessed from two baseline questions: (i) In general, at what time do you go to sleep? and (ii) In general, at what time do you wake up? Sleep duration was obtained by the difference in hours between the start and end of sleep. A cut-off point of sleep duration of 3 h or less and 15 h or more (116 students), as used by Abreu, was established to exclude implausible data [11]. The variable ‘sleep duration’ was categorized as short, adequate and long according to age group. For children of school age (6 to 12 years), sleep duration of 9 to 12 h is recommended, and for adolescents (13 to 18 years), 8 to 10 h is recommended [13].

The economic status of the participants’ families was constructed from information about durable goods at home (car, motorcycle, computer, refrigerator, freezer, washing machine, dishwasher, microwave and DVD player) and characteristics of the area of residence, such as piped water and paved streets, according to the Brazilian Economic Classification Criteria [33]. An indicator was created to represent the economic position of the families based on the methodology used by Barros and Victora [34]. The first component generated by principal component analysis (PCA), which captures the largest possible amount of data variability with a single linear combination, was retained [35, 36]. Thus, the 11 variables of durable goods at homes and residences were used in a 1-factor PCA model, which obtained an eigenvalue of 2.05 and a Cronbach alpha of 0.21 and explained 19.3% of the total data variability. Subsequently, the factorial score was divided into quintiles.

Physical activity was measured by the time accumulated in the seven days before the questionnaire, according to one of the modules used in the National School Health Survey (PeNSE) [37]. The variable was categorized as inactive (did not perform any physical activity in the last week), insufficiently active (1 to 149 min), insufficiently active (150 to 299 min) and active (more than or equal to 300 min) [37, 38].

Weight and height were measured by trained interviewers. Weight was measured with a Tetrapolar bioimpedance scale (Tanita BC-558), and height was measured twice with the AlturaExata portable stadiometer with a variation of 0.5 cm. Both procedures were performed according to Gordon’s protocol [39]. Participants were classified by BMI z-scores according to gender and age group, based on the WHO classification [40].

### Statistical analyses

First, the effect of the intervention on the outcome was analysed. Since there was no significant association between the intervention and the outcome, further analyses were treated as a prospective cohort. Although there was no specific intervention for sleep duration and CMD, interventions in food intake, physical activity and sedentary behaviour could certainly affect sleep and mental health. Therefore, preliminary analyses were also performed with only the control group to address this possibility. As the same direction of results was observed, we maintained the analysis with the complete sample (controlling the effect of the intervention). The results of the first analysis of the effect of the intervention on the outcome and the control group-only analysis can be accessed in the supplementary tables for complete results (Additional file 1: Tables S1 and S2).

To evaluate the effect of sleep duration on CMD analyses stratified by sex, we used linear mixed-effects models for repeated measures through the *MIXED* procedure from the Statistical Analysis System (SAS), version 9.4. Initially, each model included time, the baseline exposure variable and an interaction term time exposure, which allowed the evaluation of potential differences between exposure groups in response change over time. The models were adjusted for continuous age, asset indicators, physical activity, BMI and group (intervention or control). The advantage of using mixed-effects models is that they account for losses of follow-up for the outcome. This approach can handle missing values for the outcome, and no data on individuals at any time were discarded.

## Results

Of 2743 eligible school students, 2528 participated in the baseline. In the first phase of the study, 2511 students answered the GHQ-12, corresponding to 92.2% of the sample, and 2308 participated in the follow-up; therefore, there was a loss of 8.1% from the first to the second data collection date. The follow-up losses came mainly from refusals (15 students) and participant absences.

Table 1 shows the profile of the children and adolescents studied according to gender, demographic characteristics, CMD, sleep duration and covariates, such as weight status and physical activity level. There were very close proportions of boys (52%) and girls and children (56.2%) and adolescents. Almost 32% of the sample had a BMI-for-age above the recommended level (18.3% overweight and 13.4% obese) and were insufficiently active, since 43.9% reported between 1 and 149 min of physical activity per week. We also observed that 57.4% of the sample had sleep duration considered adequate. We noted that 34.3% of the sample slept less than the recommended time for the age, and girls did so more frequently (35.8%). On the other hand, boys presented higher proportions of long sleep duration (9.1%) than girls (Table 1). In general, the schoolchildren of both genders and age groups had an average sleep duration of 9.1 h (SD 2.1) (not shown in the table).

**Table 1:** Characteristics of the sample at the baseline, by sex

Variables	Total n (%)	Boys n (%)	Girls n (%)	*p*-value*
Age (years				
9 to 11	1421 (56.2)	679 (51.1)	742 (61.8)	< 0.001
12 to 17	1.107 (43.8)	649 (48.9)	458 (38.2)	
Weight status				
Thinness	80 (3.3)	58 (4.6)	22 (1.9)	< 0.001
Adequate	1.588 (65.0)	841 (65.9)	747 (64.0)	
Overweight	449 (18.3)	202 (15.8)	247 (21.1)	
Obesity	327 (13.4)	175 (13.7)	152 (13.0)	
Physical activity (min/week)				
Inactive	562 (22.4)	253 (19.2)	309 (25.9)	< 0.001
insufficiently active (1–149)	1.101 (43.9)	563 (42.8)	538 (45.1)	
insufficiently active (150–299)	469 (18.7)	284 (21.6)	185 (15.5)	
Active (> = 300)	378 (15.0)	217 (16.5)	161 (13.5)	
Sleep duration (h/day)				
Short	712 (34.3)	348 (32.9)	364 (35.8)	0.001
Adequate	1.190 (57.4)	613 (58.0)	577 (56.7)	
Long	172 (8.3)	96 (9.1)	76 (7.5)	
CMD frequency				
Yes	834 (33.2)	486 (36.9)	348 (29.2)	< 0.001
No	1.678 (66.8)	832 (63.1)	846 (70.8)	
CMD score	**mean (SD)**	**mean (SD)**	**mean (SD)**	*p*-value**
0–36	11.7 (8.1)	12.5 (8.3)	10.9 (7.8)	< 0.001

Adequate sleep duration: for children of school-age (6 to 12 years), 9 to 12 h is recommended and, for adolescents (13 to 18 years), 8 to 10 h

*CMD* Common mental disorders

*Chi-square test between variables and sex. ** Student’s T test between CMD scores, ranging from 0 to 36, and the students’ gender

The frequency of common mental disorders was 33.2% at baseline and was higher in boys (36.9%) than in girls (29.2%) in both age groups. The CMD frequency was 32.2% at follow-up, with 35.1% of boys and 29% of girls. The presence of CMD was more frequent among students with sleep duration below that recommended for their age, both in boys (39.6%) and in girls (28.1%), but girls showed no significant difference. Regarding weight status, the proportions of CMD were similar, varying in boys from 32.3% (overweight) to 39.5% (adequate). Schoolchildren considered to be active and with lower economic status had higher proportions of CMD among both sexes (Table 2).

**Table 2:** CMD frequency at baseline, according to population characteristics, by sex

Variables	CMD			
	Boys (%) *n* = 486		Girls (%) *n* = 348	
Age (years)		**p chi2**		**p chi2**
9 to 11	37.4	0.661	26.4	0.007
12 to 17	36.3		33.7	
Economic status		**p trend**		**p trend**
1° quintile	27.0	< 0.001	28.5	0.017
2° quintile	32.3		24.7	
3° quintile	34.1		26.6	
4° quintile	35.5		28.1	
5° quintile	50.6		40.3	
Weight status		**p trend**		**p trend**
Thinness	32.8	0.009	36.4	0.052
Adequate	39.5		30.2	
Overweight	32.3		27.8	
Obesity	32.6		23.0	
Physical activity (min/week)		**p trend**		**p trend**
Inactive	21.3	< 0.001	25.8	< 0.001
insufficiently active (1–149)	30.9		25.1	
insufficiently active (150–299)	43.0		34.6	
Active (> = 300)	62.7		42.9	
Sleep duration (h/day)		p chi2*		p chi2*
Short	39.6	0.012	28.1	0.479
Adequate	31.6		26.0	
Long	34.4	0.586	27.6	0.761

*Chi-square test between “short and adequate sleep duration” and “long and adequate sleep duration”, and CMD, by sex

Adequate sleep duration: for children of school-age (6 to 12 years), 9 to 12 h is recommended and, for adolescents (13 to 18 years), 8 to 10 h

**Fig. 1:**
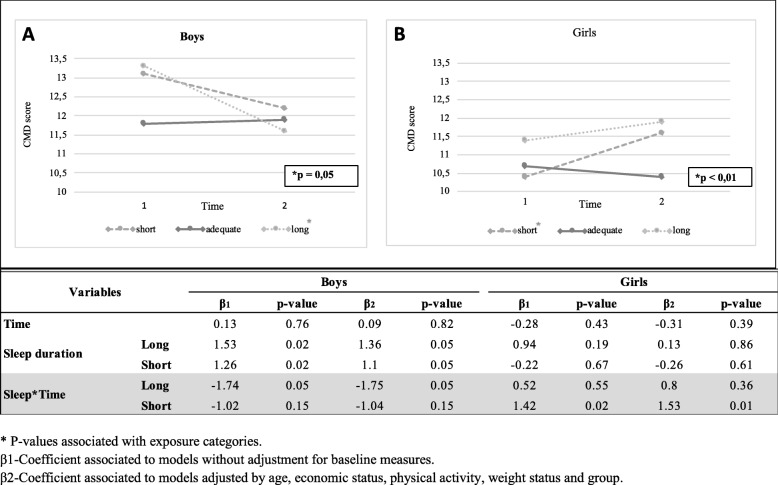
Variation in the predicted CMD score according to sleep duration, by sex, at baseline.* *P*-values associated with exposure categories. β1-Coefficient associated with models without adjustment for baseline measures. β2-Coefficient associated with models adjusted by age, economic status, physical activity, weight status and group

The average predicted values of the CMD score showed a pattern of reduction among boys and a pattern of increase among girls at the follow-up. For example, in graph B, it is possible to observe an increase in the CMD score over time among girls with short sleep duration (*p < 0.01*) (Fig. 1). The *p*-value in each graph refers to the interaction term between time and duration of sleep (time * duration of sleep). Among boys, a similar variation in the CMD score was observed in the subgroups with short sleep and long sleep, but there was a significant reduction in those with long sleep duration (*p = 0.05*).

## Discussion

The present study examined changes in CMD over time according to sleep duration in adolescents. Our findings revealed that girls who had a short sleep duration at baseline had a higher CMD score over time. Among boys, those with longer sleep had a reduction in the CMD score. These findings show different variations in CMD between the sexes.

Our results are consistent with the literature existing thus far, which suggests that short sleep duration is associated with changes in the physical and mental health of children and adolescents across a broad range of indicators of psychological, interpersonal, and somatic well-being. Typically present in common mental disorders, more fatigue, less energy, worse perception of health and symptoms such as headaches, stomach and back pain may be observed [16, 21, 41].

Among other findings, a large national sectional study of the U.S. (Youth Risk Behavior Survey data) found that young people who slept little at night were more likely to report substance use, have suicidal thoughts, and feel sad and hopeless [42]. Sarchiapone et al. [43], in a study of 11,788 young people from 11 European countries, also identified a strong association between short sleep duration and emotional and behavioural problems, especially in girls. However, because the designs of these studies were cross-sectional, it was not possible to exclude the possibility of reverse causality in such results. More recent studies have further extended these findings. A prospective, three-wave study of European and African American children evaluated the reciprocal relationships between children’s sleep and their symptoms of internalization and externalization and found that reduced sleep duration predicted greater depression, anxiety, and externalizing symptoms over time [44].

Sleep in childhood and adolescence has particularities that must be considered because they impact the duration and quality of sleep. From a biological perspective, adolescents can present delays in the sleep phases, characterized by sleeping and waking up later. There is a delay in the time of nocturnal melatonin secretion across adolescence that parallels a change in circadian phase preference from a more “morning” type to a more “evening” type, which consequently results in an increased difficulty in falling asleep at an earlier time [45, 46]. Another important factor is the altered “sleep impulse” in adolescence, in which the pressure to fall asleep accumulates more slowly than in adults, resulting in a built-up deficit across the day [15]. Added to the sleep-wake changes that flow from this biological maturation, teens experience increased academic demands and in some cases must also cope with part-time jobs, extracurricular and social activities and the intense use of electronic devices, such as cell phones, computers, video games, and television. These conflicting demands can cause insufficient sleep, which negatively affects the psychological well-being, socialization ability, and academic and work performance of adolescents [47, 48]. The American Academy of Pediatrics and The American Academy of Sleep Medicine recognize insufficient sleep in adolescents as an important public health issue, and several studies have highlighted earlier school start times (before 8:30 am) as a key modifiable contributor to short sleep duration in this population [46, 49]. A recent review of the literature about school start times, sleep, and behavioral, health and academic outcomes showed that many of the studies in support of delaying school start times have been conducted in the United States, although data from ten other countries – Hong Kong, Israel, Turkey, Norway, New Zealand, China, Switzerland, Australia, Spain, and Croatia – also show benefits in sleep when schools start later (8:30 or later) [50].

Our results are consistent with findings from other studies that assessed differences between gender and mental health. A study performed in a sleep laboratory at the Sleep Research Center in Australia exposed adolescents of both sexes to a sleepless night to test the hypothesis that their mood would be significantly worse after a night of sleep deprivation when compared to the ideal two nights’ sleep. The results showed that female adolescents presented greater vulnerability to mood deficits after sleep loss in various mood states, mainly with significant increases in depressive and anxious symptoms. Thus, the authors suggest that there is a differential sensitivity between male and female adolescents to the effects of sleep loss in some mood states [51].

Although direct comparisons are difficult due to differences in study designs, our findings corroborate the emerging literature indicating a prospective association between sleep duration and psychological outcomes in populations of similar ages. A recent longitudinal study of 3071 young people in the British Columbia Substance Use Survey (BASUS), a prospective cohort investigating the psychosocial and environmental factors associated with sleep time and two measures of depression over 12 months, showed that chronic sleep deprivation increases the risk of depression in young women [52]. A previous longitudinal study in Chicago on sleep, depressive symptoms and self-esteem data, with three years of follow-up, used latent growth models to show that students who obtained less sleep over time reported heightened levels of depressive symptoms and decreased self-esteem [48].

Some authors suggest that insufficient sleep duration itself causes physiological stress pathways to be hyperactive, and females are more stress-reactive than males due to the differential effect of testosterone on the hypothalamic-pituitary-adrenal axis. Therefore, the promotion of mental health for young people should include relevant strategies to ensure that adolescents of both sexes – especially girls, as they are the most vulnerable subgroup – can achieve the recommended amounts of sleep [53, 54].

Another important finding of our study is the frequency of baseline CMD, which corresponds to 32.2%, with a higher prevalence among boys (36.9%) than girls (29.2%). It should be emphasized that our study used a higher cut-off point, indicating more severe CMD. Brazilian multicentre data from the Study of Cardiovascular Risks in Adolescents (ERICA), with approximately 75,000 adolescents evaluated in 1248 schools, showed a prevalence of 30% of CMD among Brazilian adolescents, similar to that found in a population-based study with adolescents living in Pelotas (28.8%), a municipality in southern Brazil [8, 55]. Both Brazilian studies used the same instrument to measure CMD, but with a lower cut-off point, which indicates a higher frequency of more severe CMD in the Duque de Caxias region.

The higher prevalence of CMD among boys than among girls differs from the literature. Although these associations, particularly with the internalization of mental health outcomes, are commonly reported to be more adverse in women, men are more frequently exposed to violence, which is often linked to CMD (especially, but not exclusively, externalization problems). Thus, these results may be related to aspects of the study region itself, since these schools are located in one of the poorest municipalities of the state of Rio de Janeiro, with significant indicators of community violence. According to data from the Public Security Institute [56], of the 92 municipalities of the State of Rio de Janeiro, the Duque de Caxias region presented the third-highest number of notifications for violence against children and adolescents in 2017. Among the different types of violence, the region where the students study and live had higher rates of physical and patrimonial violence (theft and destruction or retention of personal property, among others), subtypes that occur more frequently among male adolescents. According to Curtis et al. [57], some studies have shown differences in how girls and boys relate to neighbourhood conditions, as parents generally restrict the out-of-home activities of adolescent boys less than those of girls; thus, boys may be more exposed to impoverished neighbourhood conditions. Thus, CMD seem more strongly associated with neighbourhood conditions for boys than for girls.

### Strengths and limitations

This study should be considered in the context of its strengths and limitations. Among the positive points, we highlight the longitudinal analysis of the CMD trajectory through a mixed-effects model that accounts for missing data and differences in the number of measurements per subject. Thus, in addition to providing support for the causal role of sleep loss in adolescent mental health, our findings contribute to the restricted literature that examines the differences between the sexes in the mental health response to sleep loss.

However, this study is subject to some limitations. First, sleep duration data were obtained by self-report and were not verified by objective measures such as actigraphy (a measurement of the motor activity sensor) or polysomnography (sleep study), which may lead to overestimation of sleep duration. According to some studies, such overestimation may occur not only because of the limitations of subjective sleep reports, such as memory and interpretation, but also because the simple act of going to bed may be perceived as the onset of sleep [58, 59]. Thus, we recommend evaluating not only sleep duration but also the quality of sleep and the circadian chronotype because they contribute significantly to daytime functioning, highlighting the importance of considering these factors together to better understand their impact. Second, the days of the week and weekends were not differentiated in collecting information on bedtime and wake-up time, but as the study was conducted in schools, the responses were likely related to school days.

## Conclusion

This study represents an advancement in the knowledge of child and adolescent mental health, especially the role of sleep duration in CMD changes over time. Given our findings and prior knowledge that sleep problems are common among adolescents due to maturational processes and changes in sleep, parents and adolescents should pay more attention to their sleep patterns and implement interventions if necessary. The promotion of health considering the school environment could help to change this scenario. As discussed earlier, the benefits of later school start times have gained attention in recent years, and important organizations, researchers, and educators in the field strongly recommend changes in school schedules to better attend to the biological sleep needs of adolescents owing to the social and academic demands that they experience. From the public health perspective, our findings can also contribute to discussions within the Brazilian context.

## Supplementary information

**Additional file 1: Table S1**. Variation in mean CMD over time comparing intervention with control group. β - Coefficient associated to linear mixed effects models. Group - refers to school allocation group (intervention or control). **Table S2**. Regression coefficients (β) of CMD, according to time of follow-up and sleep duration, by sex. β-Coefficient associated to linear mixed effects models only with the control group sample adjusted by age, economic status, physical activity and weight status.

## Data Availability

The participants shared their opinions and experiences upon the assurance that their confidentiality and anonymity would be protected. Hence, the research data are not publicly available because this would compromise individual privacy and our ethical approval conditions.

## Abbreviations

AASM: American Academy of Sleep Medicine
BASUS: British Columbia Substance Use Survey
BMI: Body mass index
CHA: Community health agents
CMD: Common mental disorders
ERICA: Study of Cardiovascular Risks in Adolescents
GHQ: General Health Questionnaire
PAAPAS: Parents, Students, Community Health Agents and Teachers for Healthy Eating
PCA: Principal component analysis
PDA: Personal digital assistants
PeNSE: National School Health Survey
SAS: Statistical Analysis System
WHO: World Health Organization
