# Supplementary material for: Gender differences in the impact of sleep duration on common mental disorders in school students
Source: BMC Public Health. 2020 Jan 31;20:148. doi: 10.1186/s12889-020-8260-5 (PMC6995133; doi:10.1186/s12889-020-8260-5)
Supplement: Supplementary file 1 — Additional file 1: Table S1. Variation in mean CMD over time comparing intervention with control group. β - Coefficient associated to linear mixed effects models. Group - refers to school allocation group (intervention or control). Table S2. Regression coefficients (β) of CMD, according to time of follow-up and sleep duration, by sex. β-Coefficient associated to linear mixed effects models only with the control group sample adjusted by age, economic status, physical activity and weight status. [file 12889_2020_8260_MOESM1_ESM.docx]

Supplemental Tables

Table S1 – Variation in mean CMD over time comparing intervention with control group.

β - Coefficient associated to linear mixed effects models.

Group - refers to school allocation group (intervention or control).

Table S2 - Regression coefficients (β) of CMD, according to time of follow-up and sleep duration, by sex.

β-Coefficient associated to linear mixed effects models only with the control group sample adjusted by age, economic status, physical activity and weight status.
